# The effect of a Veterans Affairs rapid rehousing and homelessness prevention program on long‐term housing instability

**DOI:** 10.1111/1475-6773.14428

**Published:** 2024-12-30

**Authors:** Alec B. Chapman, Daniel Scharfstein, Thomas Byrne, Ann Elizabeth Montgomery, Ying Suo, Atim Effiong, Christa Shorter, Sophia Huebler, Tom Greene, Jack Tsai, Lillian Gelberg, Stefan G. Kertesz, Richard E. Nelson

**Affiliations:** ^1^ IDEAS Center, Veterans Affairs Salt Lake City Health Care System Salt Lake City Utah USA; ^2^ Department of Internal Medicine University of Utah School of Medicine Salt Lake City Utah USA; ^3^ Department of Population Health Sciences University of Utah School of Medicine Salt Lake City Utah USA; ^4^ National Center on Homelessness Among Veterans Washington, DC USA; ^5^ School of Social Work Boston University Boston Massachusetts USA; ^6^ Center for Healthcare Organization and Implementation Research, Bedford VA Medical Center Bedford Massachusetts USA; ^7^ Birmingham VA Health Care System Birmingham Alabama USA; ^8^ School of Public Health University of Alabama at Birmingham Birmingham Alabama USA; ^9^ School of Public Health University of Texas Health Sciences Center San Antonio Texas USA; ^10^ Department of Family Medicine David Geffen School of Medicine at UCLA Los Angeles California USA; ^11^ Office of Healthcare Transformation and Innovation VA Greater Los Angeles Healthcare System Los Angeles California USA; ^12^ Department of Health Policy & Management Fielding School of Public Health, University of California at Los Angeles Los Angeles California USA; ^13^ UAB Heersink School of Medicine Birmingham Alabama USA

**Keywords:** homeless populations, rapid rehousing, social determinants of health, Supportive Services for Veteran Families, VA Healthcare System, Veterans

## Abstract

**Objective:**

To evaluate the effect of enrolling in Supportive Services for Veteran Families (SSVF) on short‐ and long‐term housing outcomes among Veterans experiencing housing instability.

**Study Setting and Design:**

We analyzed data from the Department of Veterans Affairs (VA) electronic health record (EHR) between October 2015 and December 2018 using the target trial emulation framework. Veterans were included in one or more trials if they were 18 years or older, had recent evidence of housing instability, had received care in VA for at least 1 year, and had never before enrolled in SSVF. Patients who enrolled in SSVF after meeting eligibility were assigned to the treatment group, while patients who did not enroll in SSVF were assigned to a control group. We extracted patients' housing outcomes from the EHR and modeled the probability of being unstably housed each day while accounting for confounders and irregular visit times.

**Data Sources and Analytic Sample:**

We extracted housing status and covariates from the VA Corporate Data Warehouse. Housing instability was ascertained using a combination of structured data elements and natural language processing.

**Principal Findings:**

We identified 238,059 unique patients who met the eligibility criteria for one or more trials. The risk of housing instability decreased in both arms over the three years after initiating a trial but was lower among SSVF enrollees, with a risk difference of −12.9% (95% confidence band −14.6%, −11.2%) on Day 120 and an attenuated difference of −2.4% (−4.0%, −0.7%) on Day 1095.

**Conclusions:**

SSVF is one of the largest rapid rehousing and homelessness prevention programs in the nation. We found that SSVF improves housing outcomes over the three years following enrollment, but the effect reduces over time. These findings can inform policy and program design for improving housing outcomes for homeless‐experienced individuals.

**What is known on this topic**

- Addressing Veteran homelessness is a high priority for the Department of Veterans Affairs.
- Supportive Services for Veteran Families (SSVF) is a large rapid rehousing and homelessness prevention program offering services to Veterans and their households experiencing housing issues.
- The overall impact of SSVF on long‐term housing outcomes has not been studied.

**What this study adds**

- SSVF reduces housing instability for up to three years after program enrollment.
- The effect is largest during the 3–12 months after entering SSVF and attenuates over time.
- SSVF is an effective approach for rapidly addressing housing instability; additional interventions may be needed to optimize long‐term housing stability.

## 1 INTRODUCTION

Homelessness is a significant public health concern. Lack of safe, stable housing has been shown to be associated with adverse health outcomes and decreased quality of life.^1^, ^2^, ^3^ In the United States, Veterans comprise a significant portion of the homeless population.^4^ The Department of Veterans Affairs (VA) has implemented several programs that assist Veterans experiencing homelessness or at risk of becoming homeless. One major program is Supportive Services for Veteran Families (SSVF), which offers various rapid rehousing and homelessness prevention services, including financial help, to individual Veterans and their households.^5^

Several studies offer evidence supporting short‐term effectiveness for particular components of SSVF (e.g., temporary financial assistance) on health and housing outcomes such as achieving and maintaining stable housing,^6^, ^7^, ^8^, ^9^, ^10^, ^11^, ^12^ showing that most Veterans enrolled in SSVF exit the program to stable housing and that temporary financial assistance leads to improved housing stability at the time of program exit and up to 1.5 years after program entry.^6^, ^10^ However, no studies have examined whether SSVF as a whole (i.e., enrolling in SSVF compared to not enrolling in SSVF) is effective in improving housing outcomes for Veterans experiencing housing instability.

Researchers and policymakers seeking to evaluate programs like SSVF are restricted by a lack of large, prospective, randomized studies with long‐term follow‐up. While randomized clinical trials (RCTs) are the gold standard study design for assessing the effect of an intervention, they are challenging to conduct for real‐world programs such as SSVF. An alternative to conducting an RCT is to emulate one using observational data such as health data recorded in the electronic health record (EHR).^13^

The objective of this study was to assess the impact of enrolling in SSVF on a Veteran's risk of being unstably housed during the three years following an episode of housing instability.

## 2 METHODS

### 2.1 Data and setting

#### 2.1.1 SSVF program and population

SSVF was developed following the Veterans' Mental Health and Other Care Improvements Act of 2008.^14^ A key distinction between SSVF and other VA homeless programs is that it operates as a partnership with local community organizations (i.e., SSVF “grantees”) across the United States. SSVF provided $379 million in funding to 378 grantees located in all 50 states and the District of Columbia in Federal Fiscal Year (FY) 2016, roughly corresponding to the start of our study, and similar amounts in FY2017–18.^15^ Through this partnership, SSVF provides supportive services that assist with achieving stable housing to Veteran households—both individuals and families—who are experiencing homelessness or at risk of losing housing. Services provided to SSVF participants include outreach and case management services; assistance in obtaining VA or public benefits; and temporary financial assistance for paying housing‐related costs. Timing and duration of SSVF services are flexible, and the amount of time a Veteran is enrolled in SSVF varies according to their particular circumstances and needs. Past studies have found a typical enrollment to be 90 days.^6^, ^10^

A key requirement for enrolling in SSVF is that a Veteran or Veteran household must be either homeless or at immediate risk of becoming homeless. The Department of Housing and Urban Development (HUD) defines four categories of homelessness^16^:

1. Literal homelessness, defined as currently lacking a “fixed, regular, and adequate nighttime residence,” and encompassing both unsheltered homelessness (e.g., sleeping on the streets) and sheltered homelessness (e.g., staying in an emergency shelter or transitional housing).
2. Imminent risk of homelessness, which includes individuals and households who will lose their primary residence within 14 days and do not have a plan for their next residence.
3. Homeless under other federal statutes, comprising youth 25 years of age or younger or families who do not meet definitions 1–2 but are considered homeless under other federal definitions.
4. Fleeing/attempting to flee domestic violence.

SSVF refers to these definitions of homelessness when determining program eligibility, requiring that a Veteran or household meet one of definitions (1), (2), or (4) to enroll in the program.^14^, ^17^ Veterans or households who meet HUD definitions of (1) or (4) qualify for rapid rehousing services, while those who meet definition (2) qualify for homelessness prevention services.

For our study, we sought to identify a population of Veterans who received care in the VA Healthcare System and met the requirements for SSVF enrollment. We were interested in both short‐ and long‐term housing outcomes. Short‐term outcomes were defined to be the probability of being unstably housed, that is, either currently being homelessness according to the HUD definition or at high risk for imminent homelessness—each day during the first 90 days following initiation of treatment, a time window corresponding to the typical length of an SSVF enrollment. Long‐term outcomes were defined to be the probability of unstable housing each day following the end of the 90‐day initial period through the end of the three‐year study period.

### 2.2 Data

We used data from the VA Corporate Data Warehouse (CDW), a database containing clinical data collected in the VA EHR, to identify patients with documentation of housing instability and to collect covariates and housing status over time. VA EHR data were linked to SSVF program data that were recorded using Homeless Management Information Systems (HMIS),^18^ a community‐based application and records system that community partners use to track SSVF program participation and outcomes. This study was approved by the University of Utah institutional review board, which waived informed consent because the research presented no more than minimal risk or harm to participants.

### 2.3 Study design

We designed our study using the target trial emulation framework, which involves specifying a hypothetical RCT investigating a question of interest and then replicating that RCT to the extent possible using observational data.^13^ The hypothetical RCT would assess the causal effect of SSVF on housing outcomes by following four steps:

1. Eligibility criteria: Patients deemed “eligible” to enroll in the RCT according to a predefined set of defined eligibility criteria would be recruited to enroll in the study.
2. Treatment assignment: Eligible patients would be randomly assigned to either enroll in SSVF and or be assigned to a non‐SSVF group that receives standard VA care.
3. Follow‐up: Housing instability would be measured regularly over a three‐year follow‐up period after entry into the RCT.
4. Comparison of outcomes: The effect of SSVF would be estimated by comparing the risk of being unstably housed at each time point of the study.

In the sections that follow, we explain how we used EHR data to emulate the hypothetical trial and estimate the effect of SSVF on housing outcomes. We constructed a sequence of 36 emulated trials between January 1, 2016, and December 1, 2018, with each new trial beginning on the first day of the calendar month. Patients were included in a trial each time they met the eligibility criteria.

#### 2.3.1 Eligibility criteria

Our cohort included Veterans who received care in VA and met the following eligibility criteria: (1) at least 18 years old; (2) evidence of housing instability in the calendar month before a trial's start date; (3) at least one year of baseline data in CDW; and (4) no previous enrollment in SSVF. These eligibility criteria were chosen to match the requirements for enrolling in SSVF^14^ and to ensure sufficient baseline data for statistical analysis. A new “patient‐trial” was created each time a patient met the eligibility criteria and was considered a single subject in the analysis (Supplement Exhibit 2).

Building off previous work,^19^, ^20^ we defined recent housing instability as having both structured and free‐text documentation of housing instability in the EHR during the month prior to a trial start date. A one‐month lookback period was used to identify episodes of housing instability that were recent enough to be considered eligible for SSVF during the first month of the subsequent trial. Both types of documentation were required to increase the specificity of the cohort definition.

The full list of values included in the structured data elements are provided in Supplement Exhibit 1. They include International Classification of Diseases (ICD)‐10 codes; a homelessness screener administered by VA providers among all outpatients; outpatient data elements indicating that a visit took place in a clinic providing homeless‐related services; inpatient data indicating the patient was treated by a provider specializing in homeless services; or administrative data recorded in the Homeless Outcomes Management and Evaluation System (HOMES) database maintained by VA to track the assessments of Veterans reporting housing instability and their use of other VA homeless programs such as U.S. Departments of Housing and Urban Development‐VA Supportive Housing (HUD‐VASH) and Grant and Per Diem (GPD).

The second form of documentation was evidence of unstable housing in free‐text clinical notes (e.g., social work notes and physician progress notes). Housing status was extracted from notes using ReHouSED, a previously validated natural language processing (NLP) system.^21^ ReHouSED assigns each note one of the following classifications: “Stably housed,” indicating that the patient is currently living in independent housing without immediate risk of housing loss; “Unstably housed,” indicating the note does not have evidence of stable housing and the patient is either currently homeless (e.g., residing in emergency shelter, living in transitional housing, or sleeping in a place not intended for human habitation), facing imminent housing loss (e.g., being evicted), or experiencing other housing‐related issues (e.g., looking for temporary housing); or “Unknown,” indicating that the note does not have documentation of current housing status.

Examples and additional details of the text extraction methods are provided in the Supplement as well as in other studies,^20^, ^21^ which have shown that ReHouSED achieves acceptable sensitivity and specificity in document classification, and that combining note classifications with structured data leads to high specificity for identifying currently unstably housed Veterans. Housing‐related visits were defined as days in which a Veteran has at least one note classified by ReHouSED as “Stably housed” or “Unstably housed.” A visit was classified as “Unstably housed” if at least half of these notes are classified as such. To be eligible for trial enrollment, at least half of housing‐related visits in a month were required to be classified as “Unstably housed.”

#### 2.3.2 Treatment assignment and time zero

For each trial, patients meeting the eligibility criteria were included in the SSVF group if they enrolled in SSVF during the calendar month when that trial started; otherwise, they were included in the No SSVF group. Time zero was defined to be the first day of that calendar month. For SSVF patients, there could be a “grace period”^13^ between enrollment in a trial and the day of the month on which the patient started treatment.

Veterans were not randomly assigned to enroll in SSVF or receive standard VA care. Instead, we assumed that treatment assignment was random conditional on observed baseline factors recorded in CDW prior to the start date of each trial in which the patient enrolled. Guided by the Gelberg‐Anderson Behavioral Model for Vulnerable Populations,^22^ we identified three sets of variables:

1. Predisposing factors including demographics such as age, biological sex, and race/ethnicity^23^; diagnosis of a mental health diagnosis; and diagnosis of a substance use disorder in the last year.
2. Enabling factors including whether the Veteran was identified as living in a rural or urban area; participation in other VA homeless programs (i.e., HUD‐VASH or GPD) in the prior month.
3. Need factors including service‐connected disability status and VA priority level^24^; VA health care costs in the last year; comorbidities summarized by the Charlson Comorbidity Index (CCI)^25^; and history of structured and free‐text documentation of housing instability.

The Gelberg‐Anderson includes race/ethnicity as a predisposing factor and reported race/ethnicity has been shown in prior studies to be associated with homelessness among Veterans^9^, ^26^; thus, we chose to include it in our analysis.^6^, ^10^

#### 2.3.3 Follow‐up and comparison of outcomes

For patients who enrolled in at least one trial, we processed all clinical notes containing keywords related to housing beginning one year prior to their first trial enrollment and three years after their final trial enrollment. Follow‐up was censored if the patient died prior to the end of the three‐year follow‐up period. Death dates were retrieved from the VA Death Ascertainment File.

Each day that a patient had a note determined to be related to housing status, we classified the patient's housing status that day as “Unstably housed” or “Stably housed” by taking the most common note classification on that day as described previously with the eligibility criteria. If a patient did not have any housing‐related notes, we considered them to not have a response on that date.

We evaluated the effect of SSVF as the difference in risk of being unstably housed between the two treatment arms. Our statistical approach for estimating the risk difference is described below.

### 2.4 Statistical analyses

#### 2.4.1 Descriptive statistics

We calculated univariate statistics for key baseline characteristics among patient‐trials. We also counted the number of housing‐related visits each month in each group and the proportion of those visits classified as “Unstably housed.” For Veterans in the SSVF group, we retrieved additional data from HMIS, including socioeconomic variables, services received during SSVF, and length of enrollment. These variables were collected as part of SSVF participation and thus not available for the non‐SSVF group.

#### 2.4.2 Sampling procedure

The VA serves a large population, and many unique patients met our eligibility criteria. Furthermore, enrolling patients in multiple trials and analyzing repeated measures for each patient‐trial could result in a large dataset that would be computationally infeasible to analyze with our available computing resources. To overcome this, patient‐trials in the No SSVF group were randomly selected to be included in the analysis with a probability of 20%. We retained all patient‐trials in the SSVF group.

#### 2.4.3 Inverse probability of treatment weighting

To adjust for differences in baseline characteristics between SSVF and No SSVF patient‐trials, we performed inverse probability of treatment weighting (IPTW) on the sampled dataset.^27^ Specifically, we modeled the probability of entering SSVF conditional on baseline characteristics using a logistic regression model that included a separate intercept for the 36 trials and was weighted by the inverse of the sampling probabilities (i.e., 1 for SSVF patient‐trials and 1/0.2 = 5 for No SSVF patient‐trials). Weights were calculated as one over the predicted probability that a patient‐trial would enroll in the patient's observed treatment group and were stabilized using predictions from a second model that only included trial‐specific intercepts. We assessed the quality of the model fit by comparing adjusted standardized mean differences (SMDs) among the complete dataset of SSVF and non‐SSVF patient‐trials for each variable in the treatment model.^28^

#### 2.4.4 Inverse intensity weighting

Housing status was measured when patients had an interaction with the VA health care system and a provider documented housing status in a note. These health care encounters occurred when the need arose instead of at regular, pre‐scheduled timepoints, which has the potential to bias effect estimates in longitudinal studies.^29^ We used inverse intensity weighting (IIW) to adjust for irregular visit times. Additional details regarding the visit intensity models and weighting procedures are given in the Supplement.

### 2.5 Outcome model

We fit a marginal structural model to estimate the treatment effect of SSVF on the probability of being unstably housed each day during the three years following trial enrollment. The outcome model was a logistic regression model with a trial‐specific intercept, time in days since trial enrollment (using cubic splines), and interactions between time and SSVF enrollment. The model was weighted by the product of inverse probability of treatment and inverse intensity weights.

Probabilities of being unstably housed were obtained each day for each of the 36 trials under each treatment, and the daily risk difference for each trial was calculated as the estimated probability of being unstably housed in the SSVF group minus the estimated probability of being unstably housed in the No SSVF group. To obtain overall risk and risk difference estimates, we pooled each of the trial‐specific estimates as a weighted average proportional to the overall number of patients within each trial in the sampled dataset.

### 2.6 Bootstrapping procedure

We used bootstrapping to calculate 95% confidence bands for risk and treatment effect estimates. Details around our bootstrapping procedure are provided in the Supplement and illustrated in Supplement Exhibit 3.

### 2.7 Subgroup analysis

As a rapid rehousing and homelessness prevention program, SSVF specifically targets Veterans who are experiencing acute issues related to homelessness, which could include those experiencing recent first‐time housing issues as well as those with a history of recurring housing instability. To assess whether the effect of SSVF differs for these two groups, we performed a subgroup analysis comparing housing instability and the treatment effect of SSVF across two groups defined by the amount of time between a patient's first structured documentation of unstable housing and trial entry. The first group included all patient‐trials whose first VA documentation of unstable housing was between 1 and 12 months prior to trial enrollment, while the second group included all patient‐trials whose first documentation was 13+ months prior to trial enrollment. We chose these subgroups based on the HUD definition of chronic homelessness of experiencing more than 12 months of housing instability.^30^

In each subgroup, we estimated the probability of housing instability for SSVF and non‐SSVF enrollees and the risk difference. We compared the two subgroups by subtracting the estimated risks and risk difference among the 1–12 months group from the 13+ months group, bootstrapping 95% confidence bands for each statistic.

## 3 RESULTS

A total of 238,059 patients with documented housing instability were enrolled in at least one trial, with 229,100 unique patients enrolled in at least one No SSVF trial and 26,822 unique patients enrolling in SSVF for a total of 702,460 patient‐trials (675,638 No SSVF, 26,822 SSVF). Patient‐trial baseline characteristics are shown in Table 1. In the Supplement, we present the count of patients who met each inclusion criterion (Supplement Exhibit 4), as well as the count in each “trial” and proportion who enrolled in SSVF (Supplement Exhibit 5).

**TABLE 1:** Baseline characteristics.

Characteristic	SSVF	No SSVF
	*N* = 26,822^a^	*N* = 675,638^a^
Demographics		
Age (mean, SD)	53 (13)	54 (13)
<40	5345 (20%)	128,947 (19%)
40–49	3612 (13%)	85,753 (13%)
50–59	8928 (33%)	202,782 (30%)
60+	8944 (33%)	258,219 (38%)
Sex		
Female	2781 (10%)	62,148 (9.2%)
Male	24,048 (90%)	613,553 (91%)
Race/Ethnicity^b^		
White	12,771 (48%)	347,308 (51%)
Black/African American	10,320 (38%)	237,901 (35%)
Hispanic or Latino	1812 (6.8%)	43,309 (6.4%)
American Indian/Alaska Native	293 (1.1%)	8403 (1.2%)
Asian	159 (0.6%)	4111 (0.6%)
Native Hawaiian/Pacific Islander	199 (0.7%)	4390 (0.6%)
Mixed Race	318 (1.2%)	8330 (1.2%)
Missing	950 (3.5%)	21,886 (3.2%)
Rurality		
Urban or Missing	23,596 (88%)	582,952 (86%)
Rural	3233 (12%)	92,749 (14%)
Co‐morbidities and health care utilization		
Charlson co‐morbidity Index	1 (2)	1 (2)
Any mental health diagnosis	18,735 (70%)	511,905 (76%)
Any substance use disorder	13,653 (51%)	375,165 (56%)
VA health care costs in prior year ($) (mean, SD)		
Outpatient	14,004 (15,022)	15,034 (16,311)
Inpatient	14,600 (39,828)	20,540 (52,816)
Pharmacy	1696 (7731)	1849 (8476)
VA benefits and program participation		
Service‐connected disability (%)		
0	16,324 (61%)	353,779 (52%)
1–99	10,154 (38%)	271,872 (40%)
100	351 (1.3%)	50,050 (7.4%)
VA priority group		
Group 1	3620 (13%)	172,865 (26%)
Groups 2–4	5952 (22%)	133,594 (20%)
Group 5	13,393 (50%)	284,812 (42%)
Groups 6–8	1954 (7.3%)	43,206 (6.4%)
Missing	1910 (7.1%)	41,224 (6.1%)
Other VA homelessness programs		
HUD‐VASH	6952 (26%)	153,890 (23%)
GPD	4434 (17%)	83,756 (12%)
Homelessness history in structured VA EHR data		
Structured documentation of unstable housing in month prior		
ICD‐10	21,364 (80%)	414,168 (61%)
Homelessness screener	1047 (3.9%)	53,411 (7.9%)
Outpatient data element	18,007 (67%)	353,008 (52%)
Inpatient provider specialty	703 (2.6%)	45,253 (6.7%)
HOMES administrative data	6153 (23%)	73,221 (11%)
Months since first structured documentation of unstable housing (mean, SD)	36 (32)	41 (33)
1	3754 (14%)	93,792 (14%)
2–6	4078 (15%)	67,406 (10.0%)
7–12	1864 (6.9%)	39,351 (5.8%)
13–36	4859 (18%)	119,061 (18%)
>36	12,274 (46%)	356,091 (53%)
Housing‐related visits processed by NLP in previous year		
Count of housing‐related visits (“Stably housed” or “Unstably housed”)	19 (19)	20 (20)
Proportion classified as unstable	0.77 (0.19)	0.72 (0.24)

*Note*: Patient characteristics at time of program entry for patients enrolled in SSVF or No SSVF emulated trials. Counts refer to the number of patient‐trials.

^a^ Number of patient‐trials (%).

^b^ Race and ethnicity were combined into a single variable such that all racial categories other than “Hispanic or Latino” refer to non‐Hispanic individuals.

Abbreviations: EHR, electronic health record; GPD, Grant and Per Diem; HUD‐VASH, Department of Housing and Urban Development‐Veterans Affairs Supportive Housing; ICD, international classification of diseases; NLP, natural language processing; SD, standard deviation; SSVF, supportive services for Veteran families; VA, Veterans Affairs.

Patients enrolling in SSVF differed from patients with housing instability who did not enroll in SSVF in terms of health care needs and homelessness history. SSVF patients tended to have fewer mental health diagnoses and health care costs than No SSVF patients. They were less likely to have a service‐connected disability and were also more likely to be enrolled in VA Priority Group 5, which comprises Veterans without a service‐connected disability who have low annual income or receive a VA pension. The two groups differed in terms of documented housing history, possibly indicating different levels of housing instability or different pathways of receiving services in VA. In particular, Veterans in the SSVF group were less likely to have had VA documentation of unstable housing earlier than three years prior to the trial start date.

After weighting by inverse probability of treatment weights, the adjusted SMDs were below 0.1 for all variables (Supplement Exhibit 6), indicating good balance of the covariates in the weighted population.^28^

Additional characteristics and services received for the SSVF enrollees are shown in Supplement Exhibit 7. Most patients were enrolled in SSVF as an individual, with 9.2% reporting having one or more children in their household and 7.7% having a spouse or partner. The median length of enrollment in SSVF was 91 days. Most enrollees received temporary financial assistance during their enrollment.

### 3.1 Visits and housing status extraction

Details about information extracted by NLP and the frequency of visits are discussed in the Supplement, including descriptive figures of note counts and proportions classified as unstably housed (Supplement Exhibit 8); note titles, authoring provider types, and examples of phrases extracted from notes corresponding to housing‐related concepts (Supplement Exhibits 9–12); and results of the visit intensity models (Supplement Exhibit 13).

### 3.2 Primary analysis

**FIGURE 1:**
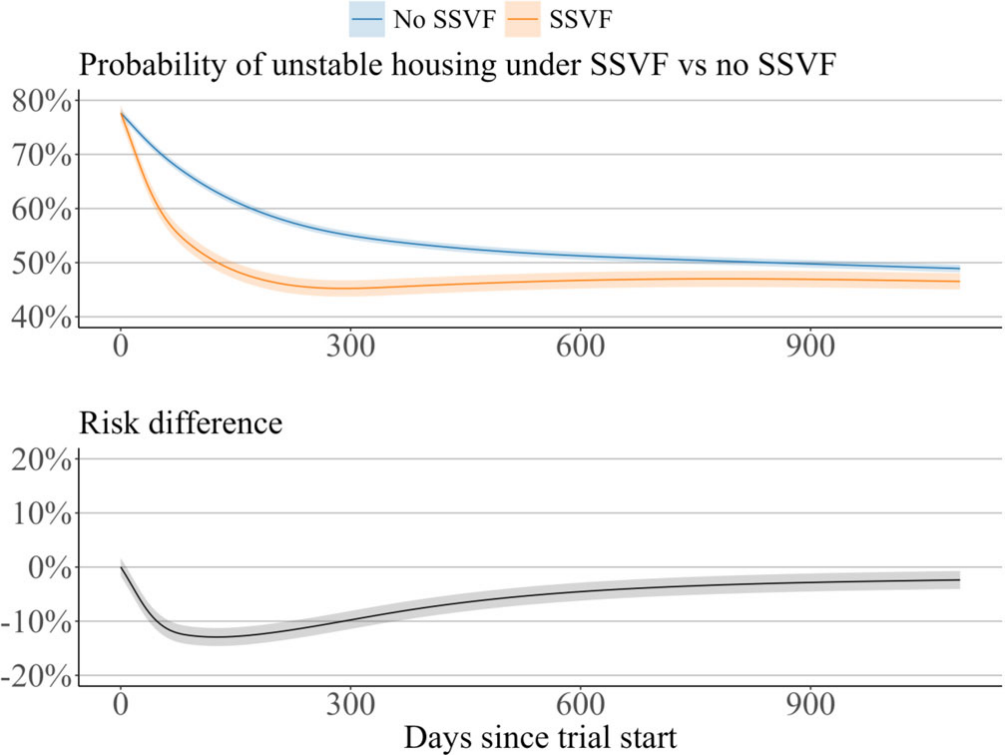
Housing instability and risk difference. Estimated risk of unstable housing under SSVF and No SSVF (top panel) and the risk difference (bottom panel) each day for up to three years after trial enrollment. 95% confidence bands are displayed around each curve.

Figure 1 shows the estimated risk of housing instability in the SSVF and No SSVF groups as well as the estimated risk difference. Housing instability declined in both groups over the three‐year period, but the decline was more rapid in the SSVF group. The difference between the two groups was largest at approximately 120 days (risk difference −12.9%; 95% confidence band −14.6%, −11.2%) and attenuated over the rest of the study period, as the estimated risk increased among the SSVF group and continued to decline among the No SSVF group. On Day 1095, the final day of the study, the risk difference was −2.4% (95% confidence band −4.0%, −0.7%).

### 3.3 Subgroup analysis

Next, we stratified the cohort according to the time between trial enrollment and their first documentation of housing instability. Patient characteristics of SSVF and No SSVF patients in each subgroup are shown in Table 2. Overall, relative to patients in the 1–12 months group, patients in the 13+ months group tended to be older and were more likely to be Black/African‐American. They had higher co‐morbidity burden, being more likely to have mental health diagnoses or substance use disorder and having higher average VA health care costs. They were more likely to be enrolled in other homelessness programs, particularly HUD‐VASH. Within the strata, differences between SSVF and No SSVF patients were similar in direction to the differences pooled across the strata (i.e., Table 1) and SMDs indicated good balance following IPTW (Supplement Exhibit 14).

**TABLE 2:** Baseline characteristics by subgroup.

	1–12 months			13+ months		
Characteristic	Overall, *N* = 210,245^a^	SSVF, *N* = 9696^a^	No SSVF, *N* = 200,549^a^	Overall, *N* = 492,285^a^	SSVF, *N* = 17,133^a^	No SSVF, *N* = 475,152^a^
Demographics						
Age (mean, SD)	52 (15)	51 (14)	52 (15)	54 (12)	54 (11)	55 (12)
<40	55,547 (26%)	2683 (28%)	52,864 (26%)	78,745 (16%)	2662 (16%)	76,083 (16%)
40–49	28,941 (14%)	1408 (15%)	27,533 (14%)	60,424 (12%)	2204 (13%)	58,220 (12%)
50–59	49,886 (24%)	2540 (26%)	47,346 (24%)	161,824 (33%)	6388 (37%)	155,436 (33%)
60+	75,871 (36%)	3065 (32%)	72,806 (36%)	191,292 (39%)	5879 (34%)	185,413 (39%)
Sex						
Female	23,318 (11%)	1312 (14%)	22,006 (11%)	41,611 (8.5%)	1469 (8.6%)	40,142 (8.4%)
Male	186,927 (89%)	8384 (86%)	178,543 (89%)	450,674 (92%)	15,664 (91%)	435,010 (92%)
Race/Ethnicity^b^						
White	116,235 (55%)	4801 (50%)	111,434 (56%)	243,844 (50%)	7970 (47%)	235,874 (50%)
Black/African American	61,699 (29%)	3332 (34%)	58,367 (29%)	186,522 (38%)	6988 (41%)	179,534 (38%)
Hispanic or Latino	15,266 (7.3%)	765 (7.9%)	14,501 (7.2%)	29,855 (6.1%)	1047 (6.1%)	28,808 (6.1%)
American Indian/Alaska Native	2450 (1.2%)	88 (0.9%)	2362 (1.2%)	6246 (1.3%)	205 (1.2%)	6041 (1.3%)
Asian	1658 (0.8%)	71 (0.7%)	1587 (0.8%)	2612 (0.5%)	88 (0.5%)	2524 (0.5%)
Native Hawaiian/Pacific Islander	1605 (0.8%)	79 (0.8%)	1526 (0.8%)	2984 (0.6%)	120 (0.7%)	2864 (0.6%)
Mixed Race	2547 (1.2%)	108 (1.1%)	2439 (1.2%)	6101 (1.2%)	210 (1.2%)	5891 (1.2%)
Missing	8743 (4.2%)	445 (4.6%)	8298 (4.1%)	14,093 (2.9%)	505 (2.9%)	13,588 (2.9%)
Rurality						
Urban or missing	176,488 (84%)	8456 (87%)	168,032 (84%)	430,060 (87%)	15,140 (88%)	414,920 (87%)
Rural	33,757 (16%)	1240 (13%)	32,517 (16%)	62,225 (13%)	1993 (12%)	60,232 (13%)
Comorbidities and health care utilization						
Charlson Comorbidity Index	1 (2)	1 (2)	1 (2)	2 (2)	1 (2)	2 (2)
Any mental health diagnosis	140,271 (67%)	6017 (62%)	134,254 (67%)	390,369 (79%)	12,718 (74%)	377,651 (79%)
Any substance use disorder	84,930 (40%)	3602 (37%)	81,328 (41%)	303,888 (62%)	10,051 (59%)	293,837 (62%)
VA health care costs in prior year ($) (mean, SD)						
Outpatient	11,215 (13,202)	11,030 (12,892)	11,224 (13,217)	16,609 (17,158)	15,688 (15,858)	16,643 (17,202)
Inpatient	13,434 (43,270)	9619 (31,245)	13,618 (43,759)	23,251 (55,576)	17,419 (43,699)	23,461 (55,946)
Pharmacy	1364 (7070)	1306 (7057)	1367 (7071)	2048 (8966)	1916 (8079)	2052 (8996)
VA benefits and program participation						
Service‐connected disability (%)						
0	108,279 (52%)	5804 (60%)	102,475 (51%)	261,824 (53%)	10,520 (61%)	251,304 (53%)
1–99	89,110 (42%)	3772 (39%)	85,338 (43%)	192,916 (39%)	6382 (37%)	186,534 (39%)
100	12,856 (6.1%)	120 (1.2%)	12,736 (6.4%)	37,545 (7.6%)	231 (1.3%)	37,314 (7.9%)
VA priority group						
Group 1	54,743 (26%)	1434 (15%)	53,309 (27%)	121,742 (25%)	2186 (13%)	119,556 (25%)
Groups 2–4	42,547 (20%)	2131 (22%)	40,416 (20%)	96,999 (20%)	3821 (22%)	93,178 (20%)
Group 5	78,366 (37%)	4450 (46%)	73,916 (37%)	219,839 (45%)	8943 (52%)	210,896 (44%)
Groups 6–8	21,744 (10%)	997 (10%)	20,747 (10%)	23,416 (4.8%)	957 (5.6%)	22,459 (4.7%)
Missing	12,845 (6.1%)	684 (7.1%)	12,161 (6.1%)	30,289 (6.2%)	1226 (7.2%)	29,063 (6.1%)
Other VA homelessness programs						
HUD‐VASH	17,202 (8.2%)	1501 (15%)	15,701 (7.8%)	143,640 (29%)	5451 (32%)	138,189 (29%)
GPD	19,915 (9.5%)	1038 (11%)	18,877 (9.4%)	68,275 (14%)	3396 (20%)	64,879 (14%)
Homelessness history in structured VA EHR data						
Structured documentation of unstable housing in month prior						
ICD‐10	121,070 (58%)	7344 (76%)	113,726 (57%)	314,462 (64%)	14,020 (82%)	300,442 (63%)
Homelessness screener	26,798 (13%)	469 (4.8%)	26,329 (13%)	27,660 (5.6%)	578 (3.4%)	27,082 (5.7%)
Outpatient data element	125,294 (60%)	6789 (70%)	118,505 (59%)	245,721 (50%)	11,218 (65%)	234,503 (49%)
Inpatient provider specialty	15,505 (7.4%)	230 (2.4%)	15,275 (7.6%)	30,451 (6.2%)	473 (2.8%)	29,978 (6.3%)
HOMES administrative data	26,470 (13%)	2416 (25%)	24,054 (12%)	52,904 (11%)	3737 (22%)	49,167 (10%)
Months since first structured documentation of unstable housing (mean, SD)	3 (5)	3 (4)	3 (5)	57 (25)	55 (25)	58 (25)
1	97,546 (46%)	3754 (39%)	93,792 (47%)	—	—	—
2–6	71,484 (34%)	4078 (42%)	67,406 (34%)	—	—	—
7–12	41,215 (20%)	1864 (19%)	39,351 (20%)	—	—	—
13–36	—	—	—	123,920 (25%)	4859 (28%)	119,061 (25%)
>36	—	—	—	368,365 (75%)	12,274 (72%)	356,091 (75%)
Housing‐related visits processed by NLP in previous year						
Count of housing‐related visits (“Stably housed” or “Unstably housed”)	12 (15)	13 (14)	12 (15)	23 (21)	22 (21)	23 (21)
Proportion classified as unstable	0.74 (0.22)	0.76 (0.20)	0.74 (0.22)	0.71 (0.24)	0.78 (0.19)	0.71 (0.24)

*Note*: Patient characteristics stratified by time since first structured documentation of housing instability in the VA EHR. Counts refer to the number of patient‐trials.

Abbreviations: EHR, electronic health record; GPD, Grant & Per Diem; HUD‐VASH, Department of Housing and Urban Development‐Veterans Affairs Supportive Housing; ICD, International Classification of Diseases; NLP, natural language processing; SD, standard deviation; SSVF, supportive services for Veteran families; VA, Veterans Affairs.

^a^ Number of patient‐trials (%).

^b^ Race and ethnicity were combined into a single variable such that all racial categories other than “Hispanic or Latino” refer to non‐Hispanic individuals.

**FIGURE 2:**
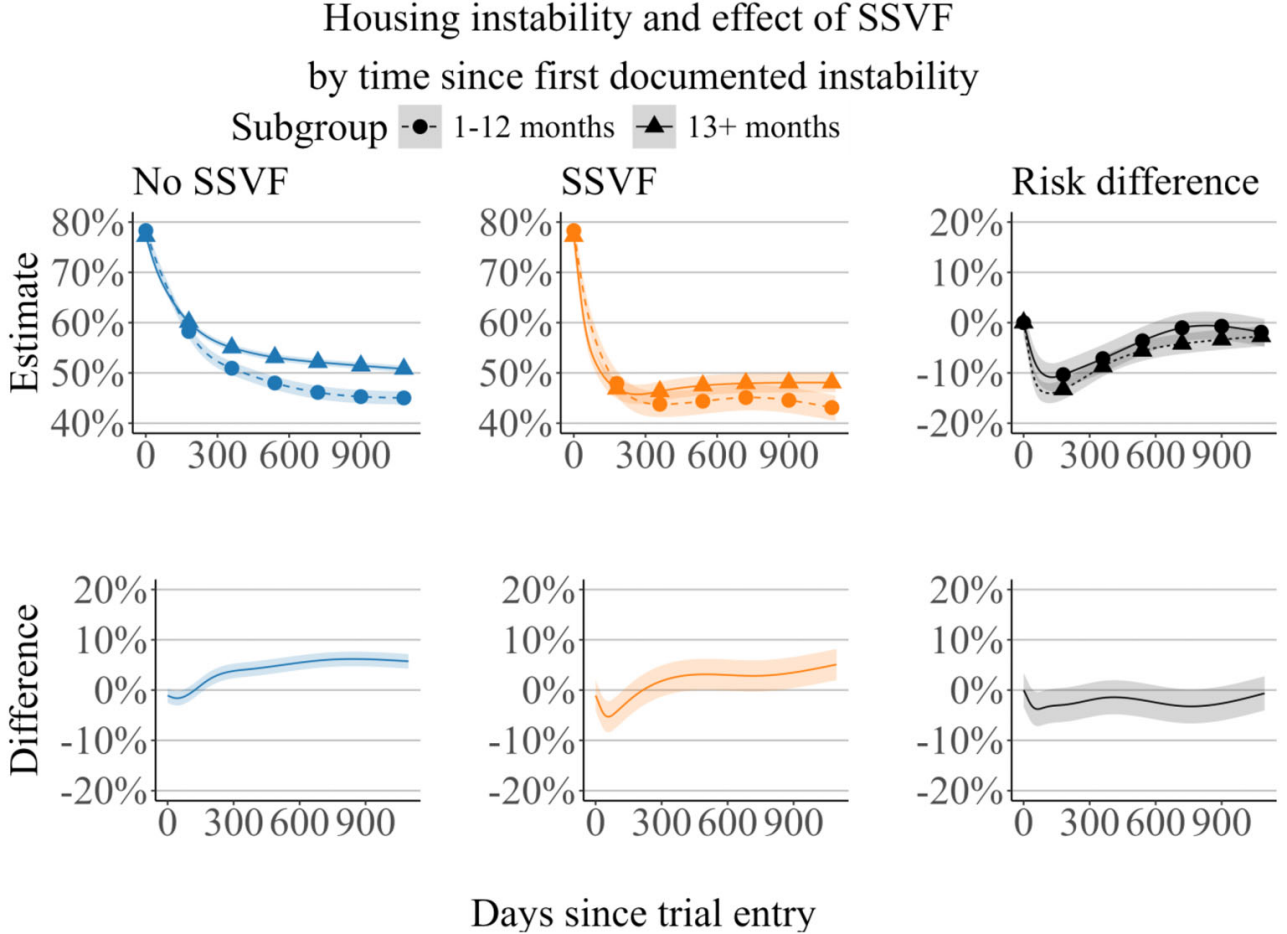
Subgroup analysis. Risk of unstable housing and risk difference by subgroups defined by time since first documentation of housing instability in VA. 95% confidence bands are displayed around each curve.

Figure 2 shows the estimated housing instability by treatment status and subgroup, the risk difference in each subgroup, and the differences between the two subgroups. During the first 90 days of follow‐up, the risk of unstable housing was lower in the 13+ month group compared with the 1–12 months group for patients enrolled in SSVF (difference − 4.5%; 95% confidence band −7.6%, −1.4%) and was similar across the two groups among patients not enrolled in SSVF. By the last day of the study, the 13+ months group had crossed over to being higher than the 1–12 months group under both SSVF (5.1%; 2.0%, 8.2%) and No SSVF (5.7%; 4.3%, 7.2%). While the levels of housing instability differed across the two subgroups, the risk difference was similar, and there was not substantial evidence for a differential treatment effect.

## 4 DISCUSSION

SSVF is one of the largest homeless prevention and rapid rehousing programs in the nation and one of the primary mechanisms through which the VA supports Veterans experiencing housing instability. Our findings suggest that SSVF reduces a Veteran's risk of housing instability, as measured based on clinical notes containing information about housing status, during the three years after they initiate SSVF. The benefit of SSVF appears to hold among patients who exhibit both recent first‐time as well as recurring patterns of housing instability and is largest after approximately three months, which aligns with the average time between SSVF entry and exit.^6^ The effect then attenuates over the remainder of the three‐year follow‐up period.

Broadly speaking, our study is important in light of the substantial expansion of rapid rehousing programs (both for Veterans and the broader population) that has occurred over the past decade,^31^ and our findings make an important contribution to the highly limited body of research that has investigated the impact of such programs.^32^ Although SSVF helps resolve housing issues for a subset of patients, the difference in housing outcomes was small toward the end of the study (e.g., 900–1095 days after trial enrollment), and the risk of unstable housing remained relatively high in both the SSVF and No SSVF groups, particularly for Veterans with a longer history of documented housing instability. The results shown here are similar to those from a previous study examining the effect of temporary financial assistance in reducing long‐term housing instability among SSVF enrollees, showing that the benefit of temporary financial assistance peaked after 45 days and attenuated thereafter.^10^

These findings have important implications for SSVF policymakers, as well as other homeless assistance programs in VA and the community. Our results suggest that SSVF, in alignment with the program's intent of providing time‐limited assistance to quickly stabilize enrollees, is an effective strategy for rapidly improving housing instability, but that additional interventions may be needed to continue to reduce housing instability over time. The few studies of rapid rehousing and homelessness prevention programs outside of VA have shown similarly mixed results with respect to the effect of these programs on long‐term stable housing.^32^, ^33^, ^34^ Taken together, this research suggests that intensive, time‐limited services may have limited impact on long‐term housing outcomes. From a policy perspective, this limited impact over the long‐term should not necessarily be perceived as a shortcoming of the program given the program's goals and relatively short episode length. Indeed, it remains an open question for policymakers about how to best evaluate the success of homelessness prevention and rapid rehousing programs in a manner that balances their short‐term nature against the desired duration of their impact.

Nonetheless, to help patients maintain long‐term stable housing, programs and treatment plans should be designed with both short‐term and long‐term outcomes in mind. VA utilizes several strategies for improving long‐term housing outcomes, including the recent Shallow Subsidy service within SSVF—which offers extended financial assistance for SSVF participants—or programs such as HUD‐VASH that offer long‐term housing support to sustain permanent housing. Future work should examine what services can improve long‐term housing outcomes in rapid rehousing and homelessness prevention programs.

Services should also be targeted to an individual's needs, with progressive engagement with high‐need Veterans to offer appropriate services over time.^35^ Considering individual patients' contextual factors is especially important when considering the potential for disparities in housing outcomes across demographic and socioeconomic groups. For example, past studies of Veteran homelessness founded differences across race/ethnicity groups in service utilization and housing outcomes.^36^, ^37^, ^38^ In our subgroup analysis, we found that Black/African‐American Veterans were overrepresented in the group with recurrent housing instability compared with the group with recent first‐time evidence of housing instability. Although the effect of SSVF was similar in both the recent first‐time and recurring unstably housed subgroups, the overall risk of housing instability was higher for patients with recurring housing instability, and individuals in this group may require more intensive or sustained services. Targeted delivery of services to high‐need Veterans experiencing housing instability should be a focus of future work.

### 4.1 Limitations

This study has several limitations. First, inclusion criteria in our study and the longitudinal outcome of housing status over time were defined using administrative and clinical data elements that are prone to measurement error.^39^, ^40^ In particular, classifications of free‐text notes using NLP depend on the precise language used by clinicians to document housing status, and our heuristic of aggregating information across notes based on whether at least half of notes during a visit were classified as unstable is not guaranteed to represent the patient's true documented housing status on that day. Adjusting for measurement error in studies of Veteran homelessness using EHR data should be the subject of future work. Second, only Veterans who used VA health care or homeless services were included in the cohort; some findings may not generalize to other Veterans or populations at risk of homelessness. Third, as with any observational study, our analysis is subject to residual confounding or selection bias. We attempted to account for this by designing our study using the target trial framework, which seeks to minimize differences between observational studies and clinical trials and utilizing multiple types of EHR data to adjust for known confounders and visit irregularity.

## 5 CONCLUSIONS

SSVF is one of the largest homeless prevention programs in the United States. Our findings suggest that SSVF improves short‐term housing outcomes for Veterans experiencing housing instability and support the program's role as one of the VA's primary mechanisms for rapid rehousing and homelessness prevention. Future work should examine interventions for optimizing long‐term housing stability (>3 years) for Veterans who enroll in SSVF.

## Supporting information


**Data S1.** Supporting information.
