# Supplementary material for: The effect of a Veterans Affairs rapid rehousing and homelessness prevention program on long‐term housing instability
Source: Health Serv Res. 2024 Dec 30;60(Suppl 3):e14428. doi: 10.1111/1475-6773.14428 (PMC12052498; doi:10.1111/1475-6773.14428)
Supplement: Supplementary file 1 — Data S1. Supporting information. [file HESR-60-0-s001.docx]

Supplemental Materials: The effect of a Veterans Affairs rapid rehousing and homelessness prevention program on long-term housing instability

# Contents

- [Methods](#_Methods)
  - [Target trial emulation approach](#_Target_trial_emulation)
    - [Supplement Exhibit 1](#exhibit1)
    - [Supplement Exhibit 2](#exhibit2)
  - [Inverse intensity weighting](#_Inverse_intensity_weighting)
  - [Bootstrapping procedure](#_Bootstrapping_procedure)
    - [Supplement Exhibit 3](#exhibit3)
- [Results](#_Results)
  - [Cohort](#_Cohort)
    - [Supplement Exhibit 4](#exhibit4)
    - [Supplement Exhibit 5](#exhibit5)
  - [Patient characteristics and IPTW](#_Patient_characteristics_and)
    - [Supplement Exhibit 6](#exhibit6)
    - [Supplement Exhibit 7](#exhibit7)
  - [Natural language processing results](#_Natural_language_processing)
    - [Supplement Exhibit 8](#exhibit8)
    - [Supplement Exhibit 9](#exhibit9)
    - [Supplement Exhibit 10](#exhibit10)
    - [Supplement Exhibit 11](#exhibit11)
    - [Supplement Exhibit 12](#exhibit12)
  - [Visit intensity model](#_Visit_intensity)
    - [Supplement Exhibit 13](#exhibit13)
  - [IPTW for subgroups](#_IPTW_for_subgroups)
    - [Supplement Exhibit 14](#exhibit14)

# Methods

## Target trial emulation approach

We identified Veterans 18 years and older who had evidence of housing instability during the month preceding enrollment. Homelessness is documented using several structured data elements (e.g., ICD-10 codes and billing data) or in free-text clinical notes (e.g., physician progress notes or social work notes).^1–3^ We implemented a two-part operational definition of housing instability that utilized both types of documentation. The full list of data elements is included in the **Supplement Exhibit 1**.

First, structured data were extracted between December 1^st^, 2015, and November 30^th^, 2018. We included the following structured data elements: ICD-10 codes; the homelessness screener clinical reminder (HSCR), which is a structured screening template intended to be administered during primary care visits annually for all Veterans and every 6 months following a positive screen; outpatient stop codes, which are administrative data indicating that a visit took place at a clinic providing homeless-related services; treating provider specialty from inpatient admissions indicating admission into a residential program (e.g., a domiciliary or residential treatment program); and recorded housing status or program entry recorded in VA HOMES, an administrative database tracking homeless services. Difference types of structured housing documentation may be indicative of different levels of housing instability or of different treatment pathways (e.g., outpatient vs inpatient services).

Different elements may also differ in their sensitivity/specificity for capturing homelessness. To reduce the number of false positives included in our cohort (e.g., individuals who were not truly homeless at the start of a trial), we required that individuals also had documentation of homelessness in clinical notes authored in the month before a trial. After excluding patients who did not meet the remaining eligibility criteria of having at least one year of baseline data or having never enrolled in SSVF, we retrieved clinical notes that contained keywords related to housing status and were authored in the month prior to a potential trial eligibility date. Housing status was extracted from these notes using a natural language processing (NLP) system called ReHouSED.^3^ ReHouSED assigns a document classification of “Stably housed”, “Unstably housed”, or “Unknown” to each note. Notes classified as “Unknown” are assumed to not contain information about current housing status and are excluded from analysis. After processing all notes in the month preceding a trial initiation, we restricted the cohort to patients who had at least one note classified as “Unstably housed” in the month prior to a trial start and, if there were multiple notes during that month classified as “Unstably” or “Stably housed”, at least half were classified as “Unstably housed.”

**Supplement Exhibit 1.** Data elements representing homelessness in the VA electronic health record and administrative databases.

|  | **Data Element** | **Values** |
| --- | --- | --- |
| **1) Structured documentation**  At least one of the following in the month before trial enrollment | ICD-10 | Z59.0: Homelessness |
|  | Homeless Screener | Positive: Has No Stable Housing |
|  | Inpatient provider specialty | 28: Homeless Compensated Work Therapy/Transitional Residence  29: Substance Abuse Compensated Work Therapy/Transitional Residence  37: Domiciliary CHV  39: General Compensated Work Therapy/Transitional Residence  85: Domiciliary  86: Domiciliary Substance Use Disorder |
|  | VA homeless program enrollment | Compensated Work Therapy/Transitional Residence  Domiciliary Care for Homeless Veterans  Grant and Per Diem (GPD)  Housing status: Homeless  HCHV Contract Emergency Residential Services |
|  | Outpatient clinic administrative data (stop codes) | 501: Homeless Mentally Ill Outreach  504: Grant & Per Diem Group  504: IPCC Medical Center Visit  508: HCHV/HCMI Group  511: Grant & Per Diem Individual  528: Telephone HCMI  529: HCV/HCMI Individual  590: Community Outreach Homeless Veterans |
| ***and***  ***2)* Free-text documentation**  At least half of visits are classified as “Unstably housed” in the month before trial enrollment | Note-level classification | “Unstably housed” |
|  | Visit-level classification | At least 1 note classified as “Unstably housed” *and* the count of “Unstably housed” notes is greater than or equal to the count of “Stably housed” notes |

**Supplement Exhibit 2** shows three examples of patients and whether they meet eligibility criteria for the first four trials (1/1/2016-4/1/2016). Patient #1 had housing instability each month from 12/1/2015 to 4/1/2016, had their first VA visit more than 1 year before the study start date, and entered SSVF on 4/1/2016. They meet the eligibility criteria for all four trials and enter the dataset as four separate patient-trials (Patient-Trial ID #1-4), entering the No SSVF arm for the first three trials and the SSVF arm for the fourth trial.

Patient #2 had housing instability documented on 12/1/2015, but because their first VA visit was less than one year before, they were not eligible to enter the trial beginning on 1/1/2016. They again had housing instability documented in January 2016 and were thus eligible to enroll on 2/1/2016. During that month they entered SSVF, so were enrolled in the SSVF arm. Although they continued to have housing instability documented, they were no longer eligible to enter future trials because they had already enrolled in SSVF. Thus, Patient #2 enters the dataset as a single Patient-Trial (#5).

Patient #3 only has housing instability documented during a single month (3/1/2016), which coincides with the month they entered SSVF. Because they did not have housing instability documented in the prior month, they are not eligible enter as an SSVF patient on 3/1/2016. They are also not eligible to enter the following month because they have already initiated SSVF. Thus, Patient #3 is not included in the analytic dataset.

**Supplement Exhibit 2.** Three examples of determining whether a Veteran is eligible for “trial” enrollment each month between 1/1/2016 and 4/1/2016.


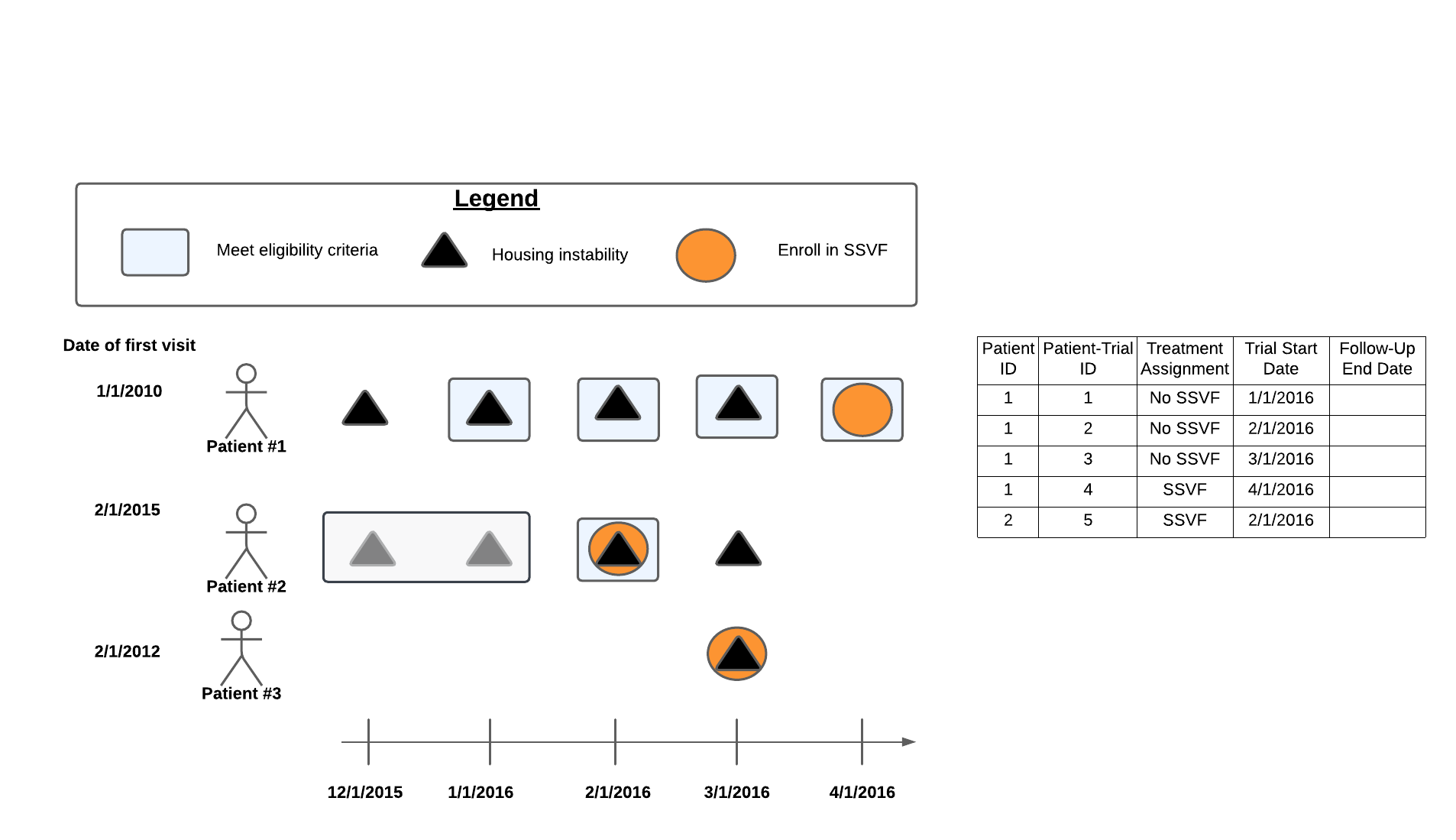


## Inverse intensity weighting

Housing status was measured when patients had an interaction with the VA healthcare system and a provider documented housing status in a note. These healthcare encounters occurred when the need arose instead of at regular, pre-scheduled timepoints, which has the potential to bias effect estimates in longitudinal studies.^4^ We used inverse intensity weighting (IIW) to adjust for irregular visit times.^5,6^ We fit a recurrent event model to estimate the intensity (i.e., instantaneous risk) of having a housing-related visit based on baseline characteristics, as well as the patient’s last observed housing status according to the NLP and whether the visit occurred after the onset of the Covid-19 pandemic, defined as March 1^st^, 2020, which we hypothesized could affect visiting frequency. Separate models were fit for the SSVF and No SSVF groups, and the baseline intensities included strata corresponding to the trial number. Inverse weights were then calculated as one over the estimated relative intensity for each visit.

## Bootstrapping procedure

Our analysis needed to account for two levels of clustering between observations in our dataset: first, clustering due to the repeated measures of housing status for each day of follow up for the same patient; and second, clustering induced by patients enrolling in multiple trials. Our bootstrapping procedure was as follows. Among the N^*^ unique patients who were included in the entire dataset in one or more trials, we sampled N^*^ patients with replacement. For each sampled patient, we retrieved all patient-trials that corresponded to that patient for each time the patient was included in the sampling with replacement. Following the sampling procedure performed in the overall analysis, we then sampled 20% of the (possibly duplicate) No SSVF patient-trials and performed the analysis using that sampled dataset. This is illustrated in **Supplement Exhibit 3**. Consider a patient who was enrolled in three trials: in the first two in the No SSVF group, and in the third one in the SSVF group (Panel A). Next, patients are sampled with replacement, and the patient is included twice in a bootstrapped (Panel B); there are now a total of six patient-trials in the initial dataset (four No SSVF, two SSVF). Finally, each of the four No SSVF entries then would have a 20% probability of being included in the bootstrapped analytic dataset (Panel C) that is used to fit the models for that bootstrap iteration.

**Supplement Exhibit 3.** Illustration of bootstrapping procedure.


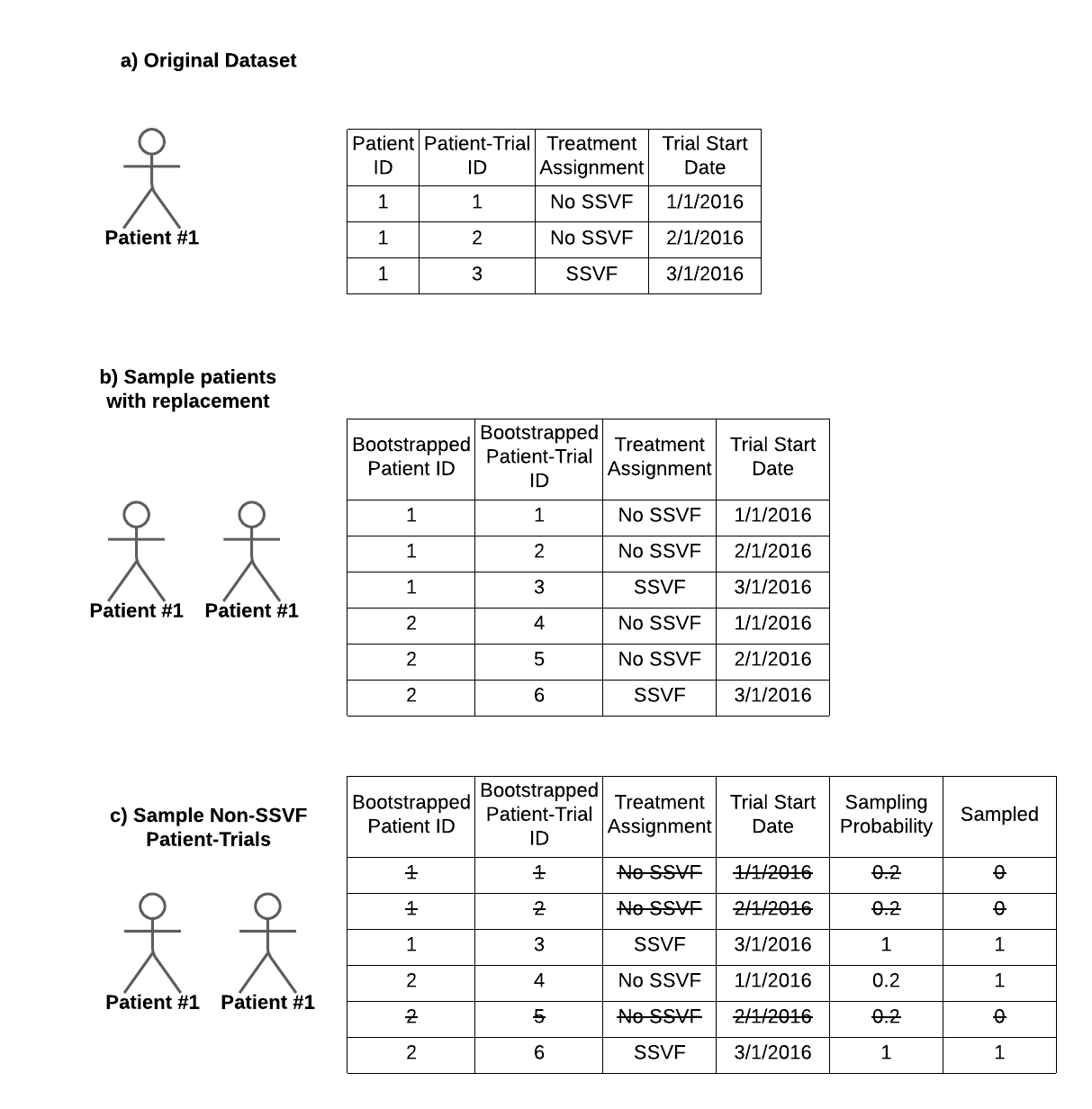


# Results

## Cohort

**Supplement Exhibit 4.** Consort diagram showing inclusion and exclusion criteria for our study.

**Supplement Exhibit 5.** Counts of patients enrolled in each emulated “trial” and the proportion of those patients assigned to SSVF.


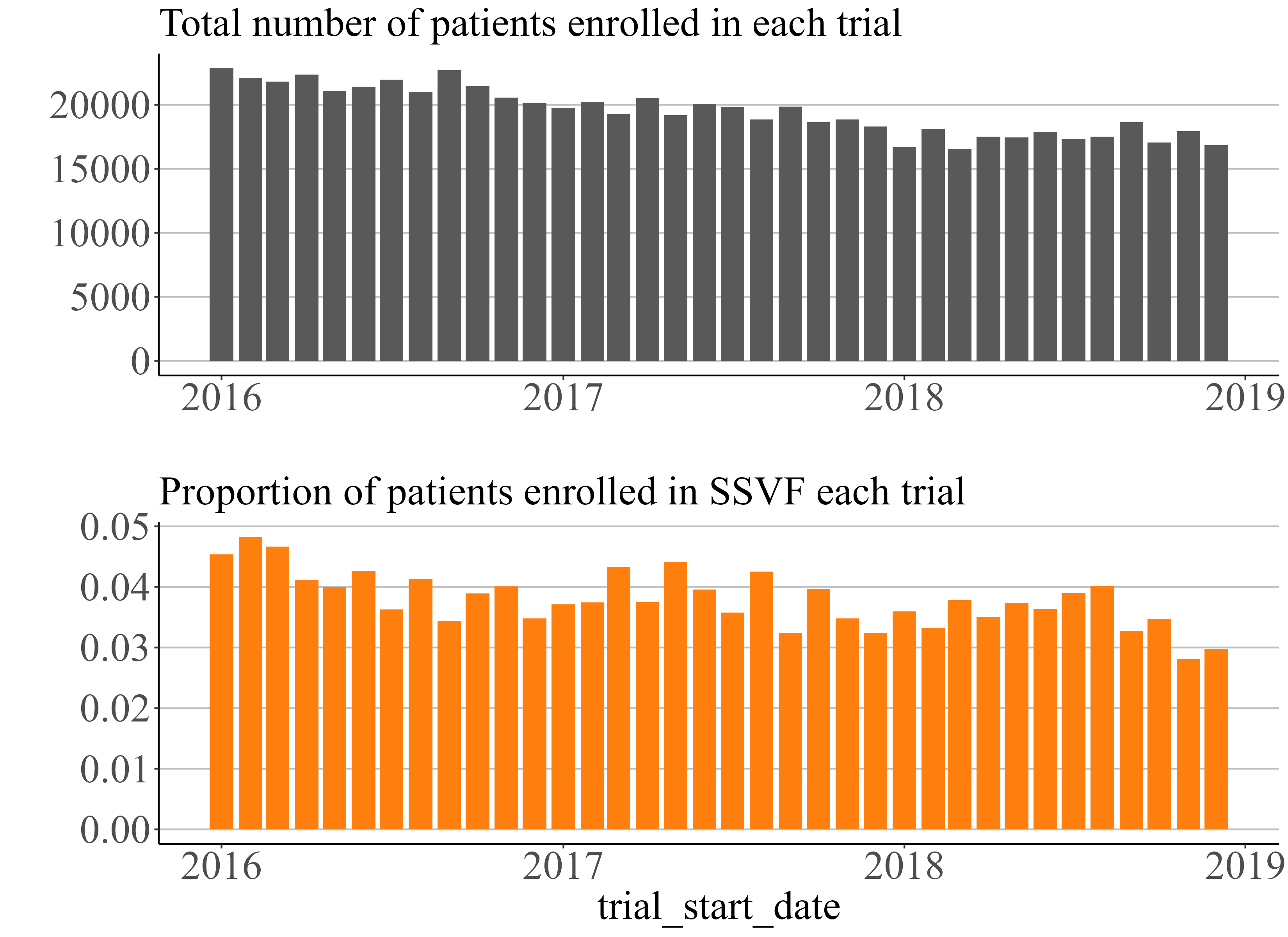


## Patient characteristics and IPTW

**Supplement Exhibit 6.** Standardized mean differences (SMDs) in variables included in the treatment model between SSVF and No SSVF patient-trials unadjusted and adjusted using inverse probability of treatment weights. Variables are ordered in descending order by the unadjusted SMD. All adjusted SMDs were below 0.1.

| **Variable** | **Unadjusted** | **Adjusted** |
| --- | --- | --- |
| prop_nlp_visits_unstable | 0.236 | 0.053 |
| homeless_icd10 | 0.183 | 0.014 |
| homeless_stop_code | 0.149 | 0.031 |
| va_inpat_cost_year_prior_std | -0.127 | 0.021 |
| priority_group_Group 1 | -0.121 | -0.015 |
| homes | 0.121 | 0.010 |
| va_inpat_cost_q_minus_1_std | -0.108 | 0.006 |
| CCI_Score | -0.087 | -0.025 |
| service_connected_0 | 0.085 | 0.009 |
| priority_group_Group 5 | 0.078 | 0.005 |
| months_since_first_homeless_cat_>36 | -0.070 | -0.013 |
| va_outpat_cost_year_prior_std | -0.066 | -0.011 |
| va_inpat_cost_q_minus_4_std | -0.066 | 0.005 |
| service_connected_100 | -0.061 | -0.007 |
| mental_health_dx | -0.059 | 0.003 |
| months_since_first_homeless_cat_2-6 | 0.052 | 0.009 |
| n_nlp_visits | -0.050 | 0.010 |
| age_cat_60+ | -0.049 | -0.026 |
| SUD | -0.046 | 0.003 |
| Homeless_Programs_GPD | 0.041 | 0.015 |
| homeless_inpat_spec | -0.041 | -0.003 |
| homeless_hscr | -0.040 | -0.003 |
| Race_White | -0.038 | -0.005 |
| age_cat_50 to 59 | 0.033 | 0.006 |
| Race_Black/African American | 0.033 | 0.003 |
| Homeless_Programs_HUD_VASH | 0.031 | -0.022 |
| service_connected_1-99 | -0.024 | -0.002 |
| priority_group_Groups 2-4 | 0.024 | 0.005 |
| va_rx_cost_year_prior_std | -0.019 | -0.003 |
| rural_Rural | -0.017 | -0.002 |
| n_nlp_visits_unstable | 0.016 | 0.021 |
| Sex_Male | -0.012 | -0.003 |
| months_since_first_homeless_cat_7-12 | 0.011 | 0.003 |
| priority_group_Missing | 0.010 | 0.002 |
| Trial_ID_2 | 0.009 | 0.009 |
| priority_group_Groups 6-8 | 0.009 | 0.003 |
| age_cat_<40 | 0.008 | 0.011 |
| age_cat_40 to 49 | 0.008 | 0.009 |
| Trial_ID_3 | 0.007 | 0.005 |
| Trial_ID_35 | -0.007 | -0.009 |
| Trial_ID_1 | 0.006 | 0.006 |
| Trial_ID_36 | -0.005 | -0.007 |
| months_since_first_homeless_cat_13-36 | 0.005 | 0.005 |
| Trial_ID_6 | 0.004 | 0.004 |
| Trial_ID_15 | 0.004 | 0.004 |
| Trial_ID_17 | 0.004 | 0.006 |
| Trial_ID_21 | -0.004 | -0.002 |
| Trial_ID_24 | -0.004 | -0.005 |
| Trial_ID_33 | -0.004 | -0.005 |
| Trial_ID_4 | 0.003 | 0.005 |
| Trial_ID_8 | 0.003 | 0.002 |
| Trial_ID_9 | -0.003 | -0.004 |
| Trial_ID_12 | -0.003 | -0.003 |
| Trial_ID_20 | 0.003 | 0.002 |
| Trial_ID_26 | -0.003 | -0.005 |
| Race_Hispanic or Latino | 0.003 | -0.001 |
| Race_Other/Missing | 0.003 | 0.003 |
| Trial_ID_5 | 0.002 | 0.003 |
| Trial_ID_7 | -0.002 | 0.000 |
| Trial_ID_19 | -0.002 | -0.001 |
| Trial_ID_23 | -0.002 | 0.000 |
| Trial_ID_28 | -0.002 | -0.002 |
| Trial_ID_34 | -0.002 | -0.003 |
| Race_American Indian/Alaska Native | -0.002 | 0.000 |
| Trial_ID_10 | 0.001 | 0.001 |
| Trial_ID_11 | 0.001 | 0.001 |
| Trial_ID_13 | -0.001 | -0.001 |
| Trial_ID_14 | -0.001 | -0.003 |
| Trial_ID_16 | -0.001 | -0.002 |
| Trial_ID_18 | 0.001 | 0.003 |
| Trial_ID_22 | 0.001 | -0.001 |
| Trial_ID_25 | -0.001 | -0.001 |
| Trial_ID_29 | -0.001 | -0.001 |
| Trial_ID_30 | -0.001 | 0.000 |
| Trial_ID_31 | 0.001 | 0.001 |
| Trial_ID_32 | 0.001 | 0.001 |
| months_since_first_homeless_cat_1 | 0.001 | -0.003 |
| Trial_ID_27 | 0.000 | -0.001 |

**Supplement Exhibit 7.** Additional patient characteristics and services received during SSVF extracted from HMIS for SSVF enrollees.

| **Characteristic** | **N = 26,822**^1^ |
| --- | --- |
| **Socioeconomic variables** |  |
| Total monthly income ($) |  |
| 0 | 8,617 (32%) |
| 1 to 499 | 3,824 (14%) |
| 500 to 1499 | 10,382 (39%) |
| 1500+ | 3,850 (14%) |
| Missing | 149 (0.6%) |
| Employment |  |
| Full-Time | 737 (2.7%) |
| Part-time | 661 (2.5%) |
| Unemployed | 24,708 (92%) |
| Missing | 2,028 (7.6%) |
| Education |  |
| College degree | 2,951 (11%) |
| Some college | 5,142 (19%) |
| High school diploma | 9,463 (35%) |
| Less than high school | 777 (2.9%) |
| Missing | 8,489 (32%) |
| Children in household (1 or more) | 2,476 (9.2%) |
| Spouse/partner | 2,072 (7.7%) |
| **Housing history** |  |
| Living situation at entry |  |
| Housed, at risk of homelessness | 3,256 (12%) |
| Sheltered | 12,971 (48%) |
| Unsheltered | 8,567 (32%) |
| Institutional | 1,683 (6.3%) |
| Missing | 345 (1.3%) |
| Times previously homeless in last 3 years |  |
| Zero times | 10,291 (38%) |
| One Time | 8,258 (31%) |
| Two times | 2,944 (11%) |
| Three times | 1,527 (5.7%) |
| Four or more times | 3,358 (13%) |
| Missing | 444 (1.7%) |
| **SSVF services** |  |
| Length of enrollment (median in days) | 91 |
| 1-30 days | 3,792 (14%) |
| 31-90 days | 9,235 (34%) |
| 91 to 180 days | 8,739 (33%) |
| 181 to 365 days | 4,312 (16%) |
| More than 1 year | 744 (2.8%) |
| Type of SSVF services |  |
| Rapid rehousing | 23,957 (89%) |
| Homelessness prevention | 2,732 (10%) |
| Other/Missing | 133 (0.5%) |
| Temporary financial assistance (any) | 15,982 (60%) |
| Rental assistance | 7,008 (26%) |
| Moving assistance | 1,728 (6.4%) |
| Security deposit | 12,056 (45%) |
| Transportation | 2,726 (10%) |
| Utilities | 4,349 (16%) |
| Non-financial supportive services (any) | 18,248 (68%) |
| Case management | 17,510 (65%) |
| Outreach | 2,110 (7.9%) |
| Assistance obtaining VA benefits | 1,008 (3.8%) |
| ^1^n (%) | |

## Natural language processing results

Our longitudinal dataset included 11,891,799 visits with documentation of housing, with a total of 15,757,715 clinical notes classified as “Stably housed” or “Unstably housed”. After duplicating visits for patients who enrolled in multiple trials, there were a total of 46,017,422 visits in our dataset. **Supplement Exhibit 8** plots the average counts of housing-related visits per patient each month in the SSVF and No SSVF groups, as well as the percent of those classified as “Unstably housed”. The frequency of visits increased leading up to the month prior to trial enrollment (in which all patients were required to have a visit with documented housing instability per the eligibility criteria) and declined over the three-year follow-up period. SSVF patients had on average more visits after trial enrollments. For both groups, the percentage of visits classified as unstable peaked in the month prior to trial enrollment, during which all patients were required to have a majority of visits classified as unstable. SSVF patients had a higher percentage of visits classified as unstable during the 1-year baseline period, but similar percentages during the first month of the trial.

**Supplement Exhibit 8.** Counts of housing-related visits each month and the proportion classified as “Unstable”.


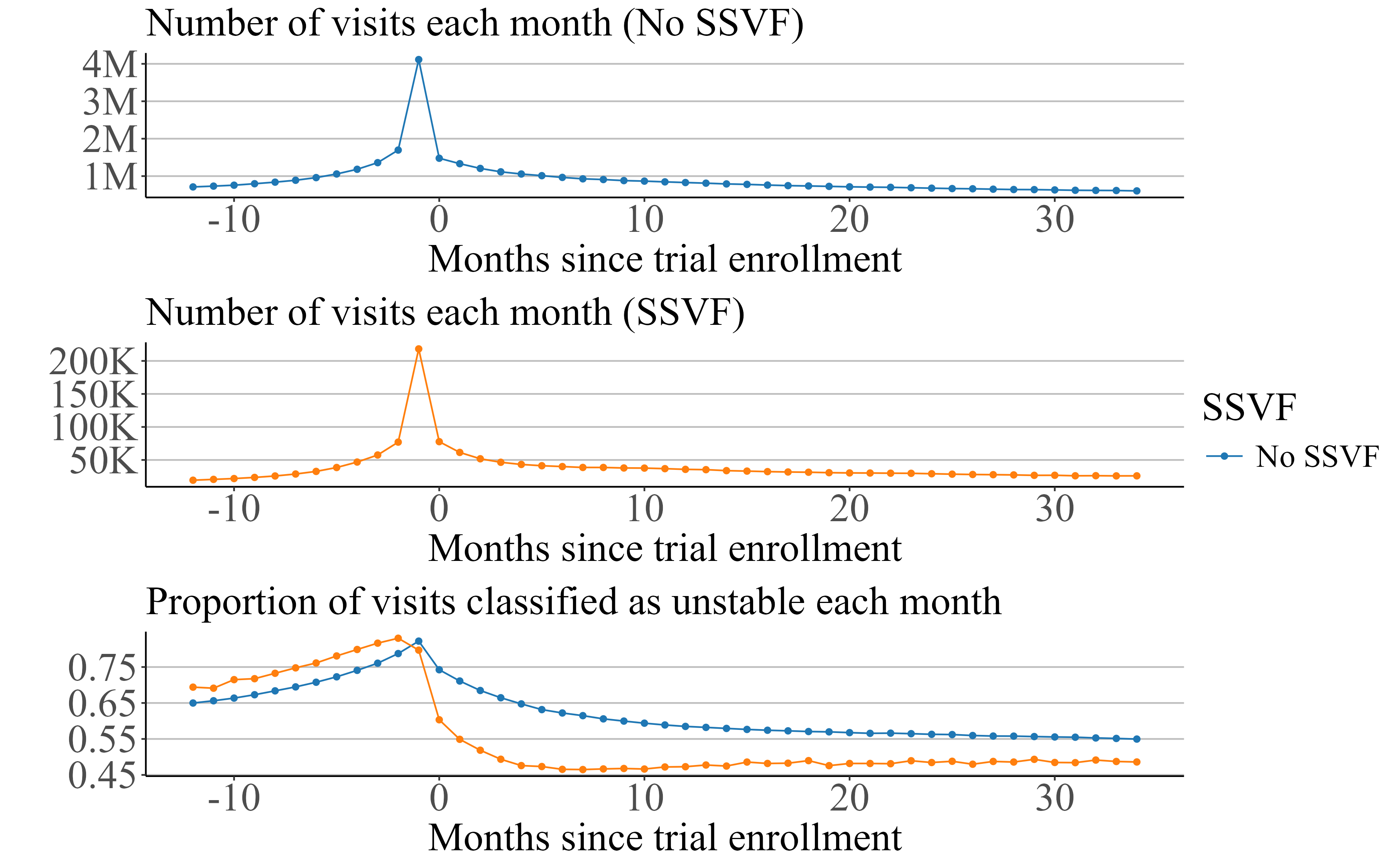


**Exhibits 9-12** display statistics describing the information extracted by NLP to classify Veteran housing status as either stably or unstably housed during the 3-year follow-up period. **Exhibit 9** shows the counts of the 15 most frequent types of authoring providers for notes containing housing documentation; **Exhibit 10** shows the most common document titles. The most common source of notes was from interactions with social workers. Nurses, counselors, psychologists, and physicians were also commonly note authors. Note titles show that interactions took place both during outpatient visits, telephone encounters, inpatient hospital stays, and in domiciliaries. **Exhibit 11** shows the most common spans of text extracted by the NLP for the concepts used to infer housing instability or housing stability. Spans are normalized by the NLP for pattern matching (e.g., “sleeps on the streets” is normalized to be “<resides> … <homeless location>”). Deidentified sentences containing evidence of housing status from a sample of notes classified as either unstably or stably housed are shown in **Exhibit 12**.

**Supplement Exhibit 9.** Most common types of authoring providers for notes classified by NLP as containing evidence of housing status.

| **Provider type** | **Count of notes** |
| --- | --- |
| Social Worker | 10,623,799 |
| Registered Nurse | 2,953,327 |
| Counselor | 1,301,700 |
| Psychiatry & Neurology | 1,298,334 |
| Resident, Allopathic (includes Interns, Residents, Fellows) | 1,280,925 |
| Psychologist | 1,063,742 |
| Nurse Practitioner | 901,308 |
| Licensed Practical Nurse | 560,577 |
| Internal Medicine | 541,847 |
| Physician/Osteopath | 475,226 |
| Pharmacist | 269,470 |
| Physician Assistant | 256,388 |
| Peer Specialist | 224,071 |
| Midwife, Lay | 201,420 |
| Emergency Medicine | 167,357 |

**Supplement Exhibit 10.** Most common titles of notes classified by NLP as containing evidence of housing status.

| **Note title** | **Count of notes** |
| --- | --- |
| Social Work Note | 1,349,801 |
| Homeless Program Note | 1,211,450 |
| Mental Health Note | 919,971 |
| Addendum | 658,390 |
| Social Work Telephone Encounter Note | 360,822 |
| Domiciliary Note | 352,869 |
| Homeless Program Social Worker Note | 347,407 |
| Psychiatry Note | 331,508 |
| Mental Health Treatment Plan Note | 327,580 |
| Social Work Case Manager Note | 325,645 |
| Mental Health Telephone Encounter Note | 273,081 |
| Mental Health Outpatient Note | 227,931 |
| Nursing Note | 194,738 |
| Discharge Summary | 193,064 |
| Telephone Encounter Note | 174,002 |

**Supplement Exhibit 11.** Counts of the most common spans of text extracted by NLP to identify evidence of stable or unstable housing.

| **Normalized span text** | **Count of notes containing span** |
| --- | --- |
| **Housing** | |
| housing | 2,641,337 |
| <his/her> residence | 1,719,401 |
| apartment | 1,316,676 |
| home visit | 813,144 |
| rent | 866,706 |
| **Homelessness** | |
| homeless | 4,883,532 |
| <resides> ... <homeless_location> | 503,959 |
| sleep in <homeless_location> | 352,363 |
| lack of housing | 289,416 |
| chronic homelessness | 196,818 |
| **Temporary housing** | |
| xxxx house | 1,124,267 |
| the domiciliary | 831,090 |
| shelter | 1,005,652 |
| domiciliary | 770,423 |
| transitional housing | 484,455 |
| **Risk of homelessness** | |
| evicted | 228,464 |
| housing | 254,856 |
| unstably housed | 204,598 |
| z59.8 | 225,848 |
| economic problem | 201,186 |
| **Doubling up** | |
| couch surfing | 35,697 |
| doubling up | 19,395 |
| **VA housing** | |
| <va_service> housing | 69,042 |
| va supported housing | 56,082 |
| subsidized housing | 43,218 |
| <patient> <va_service> <residence> | 530 |

**Supplement Exhibit 12.** Sampled sentences from notes classified by NLP as unstably or unstably housed.

| **Document classification** | **Sampled text snippets** |
| --- | --- |
| Unstably housed | “The writer called Veteran for homeless consult.”  “Veteran is also homeless.”  “She should be getting a car soon and wishes to move out of the shelter.”  “Goals: Obtaining and maintaining permanent housing.”  “Veteran states he has been living in a hotel.”  “Veteran is looking for assistance with housing.” |
| Stably housed | “Veteran is a participant in the HUD-VASH Program utilizing his housing choice voucher.”  “Living situation: Stable housing”  “He reports keeping his apartment clean and well-organized.”  “He discussed how hard he has worked over the last 2 years to get from being homeless to having his own place.”  “Resides at <APARTMENT COMPLEX>. History of homelessness.” |

## Visit intensity

The estimated coefficients (with 95% confidence intervals) for the visit intensity models are displayed in **Supplement Exhibit 13.** Factors associated with increased probability of having a visit included enrollment priority group; a longer history of documented homelessness; being classified as unstably housed during the last visit; and mental health/substance use disorders. Factors negatively associated with visiting included rurality; positive evidence of housing instability through the homelessness screener; and the visit occurring after March 2020.

**Supplement Exhibit 13.** Coefficients and 95% confidence intervals for the visit intensity model in the SSVF and No SSVF groups.

|  | **SSVF** | | | **No SSVF** | | |
| --- | --- | --- | --- | --- | --- | --- |
| **Variable** | **Intensity ratio** | **95% CI** | | **Intensity ratio** | **95% CI** | |
| Age:40 to 49 | 1.045 | 1.007 | 1.085 | 1.023 | 1.004 | 1.043 |
| Age:50 to 59 | 1.155 | 1.119 | 1.193 | 1.115 | 1.096 | 1.133 |
| Age:60+ | 1.145 | 1.107 | 1.183 | 1.071 | 1.053 | 1.089 |
| CCI Score | 1.018 | 1.013 | 1.024 | 1.018 | 1.015 | 1.021 |
| Homeless_Programs:GPD | 0.994 | 0.967 | 1.021 | 1.030 | 1.014 | 1.046 |
| Homeless_Programs:HUD-VASH | 1.237 | 1.208 | 1.267 | 1.227 | 1.210 | 1.245 |
| Male | 0.951 | 0.920 | 0.984 | 0.990 | 0.972 | 1.007 |
| Mental health diagnosis | 1.201 | 1.169 | 1.233 | 1.210 | 1.192 | 1.228 |
| Months since first homelessness:>36 | 1.354 | 1.296 | 1.414 | 1.609 | 1.571 | 1.648 |
| Months since first homelessness:13-36 | 1.227 | 1.172 | 1.285 | 1.401 | 1.366 | 1.438 |
| Months since first homelessness:2-6 | 1.261 | 1.203 | 1.321 | 1.421 | 1.381 | 1.461 |
| Months since first homelessness:7-12 | 1.165 | 1.103 | 1.231 | 1.312 | 1.272 | 1.354 |
| NLP: Count of housing-related visits in previous year | 1.018 | 1.015 | 1.020 | 1.019 | 1.018 | 1.021 |
| NLP: Count of housing-related visits in previous year classified as "Unstable" | 0.991 | 0.988 | 0.994 | 0.992 | 0.990 | 0.994 |
| NLP: count of visits in previous year: proportion "Unstable" | 1.328 | 1.229 | 1.435 | 1.212 | 1.164 | 1.262 |
| NLP: Previous housing-related visit classified as "Unstable" | 1.333 | 1.316 | 1.351 | 1.457 | 1.448 | 1.467 |
| Post-Covid | 0.774 | 0.744 | 0.806 | 0.763 | 0.749 | 0.777 |
| Race:American Indian/Alaska Native | 1.051 | 0.958 | 1.152 | 1.047 | 1.001 | 1.095 |
| Race:Black/African American | 0.925 | 0.905 | 0.945 | 0.979 | 0.968 | 0.990 |
| Race:Hispanic or Latino | 0.970 | 0.931 | 1.012 | 0.973 | 0.951 | 0.996 |
| Race:Other/Missing | 0.950 | 0.908 | 0.994 | 0.948 | 0.927 | 0.971 |
| Rural | 0.954 | 0.924 | 0.985 | 0.873 | 0.860 | 0.887 |
| Service-connected %:1-99 | 0.991 | 0.946 | 1.039 | 0.945 | 0.923 | 0.967 |
| Service-connected %:100 | 1.012 | 0.909 | 1.127 | 1.060 | 1.027 | 1.095 |
| Structured documentation of homelessness:HOMES | 1.176 | 1.145 | 1.207 | 1.233 | 1.213 | 1.253 |
| Structured homelessness:HSCR | 0.986 | 0.926 | 1.050 | 0.884 | 0.861 | 0.908 |
| Structured homelessness:ICD-10 | 1.157 | 1.123 | 1.192 | 1.203 | 1.188 | 1.217 |
| Structured homelessness:Inpatient provider specialty | 1.214 | 1.147 | 1.284 | 1.312 | 1.286 | 1.338 |
| Structured homelessness:Stop code | 0.979 | 0.957 | 1.001 | 1.015 | 1.002 | 1.027 |
| Substance use disorder | 1.174 | 1.147 | 1.202 | 1.243 | 1.227 | 1.258 |
| VA inpatient costs in previous year | 0.987 | 0.969 | 1.005 | 0.975 | 0.963 | 0.987 |
| VA inpatient costs in quarter -1 | 1.017 | 0.999 | 1.034 | 1.003 | 0.993 | 1.013 |
| VA inpatient costs in quarter -4 | 1.005 | 0.992 | 1.019 | 1.011 | 1.004 | 1.018 |
| VA outpatient costs in previous year | 1.065 | 1.054 | 1.077 | 1.051 | 1.040 | 1.063 |
| VA pharmacy costs in previous year | 0.990 | 0.980 | 1.000 | 0.992 | 0.987 | 0.997 |
| VA priority group:Group 5 | 1.182 | 1.119 | 1.249 | 1.181 | 1.150 | 1.212 |
| VA priority group:Groups 2-4 | 1.130 | 1.089 | 1.172 | 1.160 | 1.140 | 1.180 |
| VA priority group:Groups 6-8 | 1.084 | 1.017 | 1.156 | 1.097 | 1.061 | 1.134 |
| VA priority group:Missing | 1.067 | 0.995 | 1.143 | 1.041 | 1.006 | 1.078 |

## IPTW for subgroups

**Supplement Exhibit 14.** Standardized mean differences (SMDs) in variables included in the treatment model between SSVF and No SSVF patient-trials unadjusted and adjusted using inverse probability of treatment weights, stratified by time since first structured documentation of housing instability. All adjusted SMDs were below 0.1.

|  | **1-12 months** | | **13+ months** | |
| --- | --- | --- | --- | --- |
| **Variable** | **Unadjusted** | **Adjusted** | **Unadjusted** | **Adjusted** |
| CCI_Score | -0.064 | -0.013 | -0.081 | -0.016 |
| Homeless_Programs_GPD | 0.013 | 0.009 | 0.062 | 0.017 |
| Homeless_Programs_HUD_VASH | 0.077 | 0.008 | 0.027 | -0.036 |
| Race_American Indian/Alaska Native | -0.003 | 0.001 | -0.001 | 0 |
| Race_Black/African American | 0.053 | 0.01 | 0.03 | 0.002 |
| Race_Hispanic or Latino | 0.007 | -0.002 | 0 | 0 |
| Race_Other/Missing | 0.004 | 0.001 | 0.001 | 0.002 |
| Race_White | -0.06 | -0.01 | -0.031 | -0.005 |
| SUD | -0.034 | 0 | -0.032 | 0.007 |
| Sex_Male | -0.026 | -0.006 | -0.001 | -0.005 |
| Trial_ID_1 | 0.006 | 0.005 | 0.007 | 0.006 |
| Trial_ID_10 | 0.001 | 0.004 | 0 | 0 |
| Trial_ID_11 | 0 | 0 | 0.003 | 0.001 |
| Trial_ID_12 | -0.001 | -0.002 | -0.004 | -0.004 |
| Trial_ID_13 | -0.001 | 0 | 0 | 0 |
| Trial_ID_14 | 0 | 0.001 | -0.001 | -0.003 |
| Trial_ID_15 | 0.003 | 0.006 | 0.004 | 0.003 |
| Trial_ID_16 | -0.001 | -0.003 | 0 | -0.002 |
| Trial_ID_17 | 0.005 | 0.006 | 0.004 | 0.005 |
| Trial_ID_18 | 0.002 | 0.003 | 0.001 | 0.004 |
| Trial_ID_19 | -0.003 | -0.002 | -0.001 | -0.001 |
| Trial_ID_2 | 0.005 | 0.007 | 0.01 | 0.009 |
| Trial_ID_20 | 0.003 | 0.002 | 0.003 | 0.002 |
| Trial_ID_21 | -0.006 | -0.005 | -0.004 | -0.002 |
| Trial_ID_22 | 0.002 | 0 | 0.001 | 0 |
| Trial_ID_23 | -0.002 | -0.003 | -0.002 | 0 |
| Trial_ID_24 | -0.004 | -0.006 | -0.004 | -0.005 |
| Trial_ID_25 | 0.001 | 0 | -0.003 | -0.002 |
| Trial_ID_26 | -0.004 | -0.006 | -0.003 | -0.005 |
| Trial_ID_27 | 0 | -0.002 | 0 | -0.001 |
| Trial_ID_28 | -0.001 | -0.001 | -0.003 | -0.001 |
| Trial_ID_29 | -0.001 | -0.001 | 0 | -0.001 |
| Trial_ID_3 | 0.01 | 0.008 | 0.006 | 0.005 |
| Trial_ID_30 | -0.002 | 0.002 | -0.001 | 0 |
| Trial_ID_31 | 0.001 | 0 | 0 | 0.002 |
| Trial_ID_32 | 0.002 | 0.002 | 0.001 | 0.001 |
| Trial_ID_33 | -0.006 | -0.007 | -0.003 | -0.003 |
| Trial_ID_34 | -0.003 | -0.002 | -0.002 | -0.002 |
| Trial_ID_35 | -0.006 | -0.006 | -0.008 | -0.009 |
| Trial_ID_36 | -0.004 | -0.003 | -0.006 | -0.008 |
| Trial_ID_4 | 0.003 | 0.004 | 0.002 | 0.004 |
| Trial_ID_5 | 0.001 | 0.002 | 0.002 | 0.002 |
| Trial_ID_6 | 0.004 | 0.002 | 0.004 | 0.005 |
| Trial_ID_7 | -0.002 | 0 | -0.002 | 0 |
| Trial_ID_8 | 0.002 | 0.002 | 0.003 | 0.002 |
| Trial_ID_9 | -0.005 | -0.006 | -0.003 | -0.002 |
| age_cat_40 to 49 | 0.008 | 0.003 | 0.006 | 0.008 |
| age_cat_50 to 59 | 0.026 | 0.006 | 0.046 | 0.006 |
| age_cat_60+ | -0.047 | -0.028 | -0.047 | -0.023 |
| age_cat_<40 | 0.013 | 0.02 | -0.005 | 0.009 |
| homeless_hscr | -0.083 | -0.015 | -0.023 | 0.003 |
| homeless_icd10 | 0.19 | 0.022 | 0.186 | 0.005 |
| homeless_inpat_spec | -0.052 | -0.005 | -0.035 | -0.004 |
| homeless_stop_code | 0.109 | 0.018 | 0.161 | 0.03 |
| homes | 0.129 | 0.014 | 0.115 | 0.01 |
| mental_health_dx | -0.049 | 0.004 | -0.052 | 0.003 |
| months_since_first_homeless_cat_1 | -0.081 | -0.029 | -- | -- |
| months_since_first_homeless_cat_2-6 | 0.084 | 0.018 | -- | -- |
| months_since_first_homeless_cat_7-12 | -0.004 | 0.011 | -- | -- |
| months_since_first_homeless_cat_>36 | | | -0.033 | -0.013 |
| n_nlp_visits | 0.045 | 0.017 | -0.038 | 0.002 |
| n_nlp_visits_unstable | 0.061 | 0.02 | 0.042 | 0.018 |
| p_ssvf | 0.677 | 0.059 | 0.693 | 0.036 |
| priority_group_Group 1 | -0.118 | -0.028 | -0.124 | -0.007 |
| priority_group_Group 5 | 0.09 | 0.019 | 0.078 | 0 |
| priority_group_Groups 2-4 | 0.018 | 0.008 | 0.027 | 0.006 |
| priority_group_Groups 6-8 | -0.001 | -0.002 | 0.009 | 0.002 |
| priority_group_Missing | 0.01 | 0.003 | 0.01 | 0 |
| prop_nlp_visits_unstable | 0.092 | 0.003 | 0.305 | 0.068 |
| rural_Rural | -0.034 | -0.002 | -0.01 | -0.004 |
| service_connected_0 | 0.088 | 0.021 | 0.085 | 0.002 |
| service_connected_1-99 | -0.036 | -0.006 | -0.02 | 0.001 |
| service_connected_100 | -0.051 | -0.014 | -0.065 | -0.003 |
| va_inpat_cost_q_minus_1_std | -0.109 | 0.096 | -0.1 | 0 |
| va_inpat_cost_q_minus_4_std | -0.026 | -0.006 | -0.068 | 0.023 |
| va_inpat_cost_year_prior_std | -0.105 | 0.078 | -0.12 | 0.02 |
| va_outpat_cost_year_prior_std | -0.015 | -0.02 | -0.058 | -0.001 |
| va_rx_cost_year_prior_std | -0.009 | 0.002 | -0.016 | -0.011 |

# References

1. Tsai J, Szymkowiak D, Jutkowitz E. Developing an operational definition of housing instability and homelessness in Veterans Health Administration’s medical records. *PLoS One*. 2022;17(12):e0279973. doi:10.1371/journal.pone.0279973

2. Chapman AB, Cordasco K, Chassman S, et al. Assessing longitudinal housing status using Electronic Health Record data: a comparison of natural language processing, structured data, and patient-reported history. *Front Artif Intell*. 6:84. doi:10.3389/FRAI.2023.1187501

3. Chapman AB, Jones A, Kelley AT, et al. ReHouSED: A novel measurement of Veteran housing stability using natural language processing. *J Biomed Inform*. 2021;122:103903. doi:10.1016/j.jbi.2021.103903

4. Pullenayegum EM, Scharfstein DO. Randomized Trials with Repeatedly Measured Outcomes: Handling Irregular and Potentially Informative Assessment Times. *Epidemiol Rev*. 2022;44(17):121-137. doi:10.1093/epirev/mxac010

5. Lin H, Scharfstein DO, Rosenheck RA. Analysis of longitudinal data with irregular, outcome-dependent follow-up. *J R Stat Soc Series B Stat Methodol*. 2004;66(3):791-813. doi:10.1111/j.1467-9868.2004.b5543.x

6. Bůžková P, Lumley T. Longitudinal Data Analysis for Generalized Linear Models with Follow-up Dependent on Outcome-Related Variables. *Academy of Management Review*. 2006;31(2):386-408.
